# Different Effects of Regulatory Genes (*tat*, *nef*) of Human Immunodeficiency Virus Type 1 (HIV-1) on the Proliferation and Differentiation of Mouse Embryonic Stem Cells *in vitro*

**Published:** 2008-03

**Authors:** E. S. Manuilova, E. L. Arsenyeva, N. V. Khaidarova, I. M. Shugurova, L. S. Inozemtseva, V. Z. Tarantul, I. A. Grivennikov

**Affiliations:** *Department of Viral and Cellular Molecular Genetics, Institute of Molecular Genetics, Russian Academy of Sciences, Moscow, Russia*

**Keywords:** embryonic stem cells, regulatory genes of human immunodeficiency virus, transfection, embryoid bodies, proliferation, differentiation

## Abstract

To examine the effects of the *tat* and *nef* regulatory genes of human immunodeficiency virus (HIV-1) on cell differentiation we used the mouse embryonic stem cells (ESC) as a model. Proliferation, embryoid bodies (EB) formation and subsequent differentiation into cardiomyocytes, glial and neuronal cells were investigated in ESC lines transfected with these genes. It has been shown that the transfection of ESC by the *tat* gene increased their proliferating activity, whereas the *nef* gene transfected ESC showed its decrease. The number of embryoid bodies formed was higher in the cultures of ESC transfected by the *nef* and lower in the cells transfected by the *tat* in comparison with controls. The percentage of embryoid bodies with contracting cardiomyocytes was higher against control in the *nef* transfected cells and lower in ESC transfected with the *tat*. There were no reliable differences in the appearance of glial cells between control and the *nef* and *tat* transfected cell lines. Spontaneous differentiation of ESC into neuronal cells was almost not observed in the *nef* transfected cells, in contrast to control and the *tat* transfected cells. However, addition of retinoic acid (RA) to the *nef* transfected cells caused even a slight increase in neuron formation as compared to control ESC treated with RA. Thus, for the first time we have shown that the *tat* and *nef* regulatory genes of HIV-1 had a visible effect on proliferation of ESC and some first steps of their differentiation. In general, the reverse correlation between the effects of these two viral genes on ESC proliferation and differentiation were observed.

## INTRODUCTION

Studies on functional anatomy of human immunodeficiency virus (HIV) genome is one of approaches to understanding fine mechanisms of the viral interaction with cells. To fully understand functions of particular viral genes, it seems useful to investigate not only their effect on HIV sensitive human cells but also on other cell types and on cells of other species. In this respect, murine embryonic stem cells (ESC) might be a convenient model allowing one to study the impact of particular HIV genes also on the very early stages of differentiation processes. This point hasn’t yet been studied in detail, although there is some evidence of HIV and some of its genes interference with differentiation of various cell types (1, 2, 3).

Pluripotent ESC is often used as a model for the study of differentiation processes. These cells retain the ability to differentiate both *in vitro* system and after introduction in blastocytes (4). One of the main features of ESC *in vitro* is their ability to differentiate into embryoid bodies (EBs) under specific culture condition. ESC differentiation can be either spontaneous or induced by some chemical compounds and growth factors. It is well known that activin A and transforming growth factor beta-1 (TGFβ-1) induce the mesodermal cell differentiation into cardiomyocytes and skeletal muscle cells. The factors of another group – retinoic acid, fibroblast growth factor (FGF) and epidermal growth factor (EGF) induce a direct differentiation of ESC to mesodermal and ectodermal cells. Finally, nerve growth factor (NGF) and hepatocyte growth factor (HGF) are involved in the development of all three embryonic germ layers: their action causes the formation of liver, pancreas, muscular, hematopoietic and neuronal cells (5, 6, 7). Genetic control studies of the myogenic and epithelial differentiation of ESC under the action of some growth factors *in vitro* showed that the expression of tissue-specific genes was the same as in the normal developmental process (5, 8).

The another way to change proliferation and differentiation of ESC is connected to their genetic modification by the genetic vectors containing some cellular genes. It has been shown, for example, that overexpression of telomerase in ESC confers growth advantage, stress resistance, and enhanced differentiation of these cells towards the hematopoietic lineage (9).

Thus, ESC is a convenient test-system for the investigation of the effects of different genes on the processes of the cell differentiation. The present study is undertaken to investigate the effects of the HIV-1 *tat* and *nef* genes on the proliferation and differentiation of ESC. The function of these accessory viral genes is studied for a long time.

Tat protein is expressed early in the viral life cycle and stimulates viral gene expression by interacting with the TAR element found in HIV mRNA (10). Tat has been shown to bind several intracellular and extracellular proteins which suggests that it is capable of modulating various biological effects. Nef protein is an HIV-1 virulence factor shown to promote viral pathogenicity by altering host cell signaling pathways (11, 12). This protein contributes to the replication of primate lentiviruses by altering the trafficking of cellular proteins involved in adaptive immunity (class I and II major histocompatibility complex) and viral transmission (CD4 and DC-SIGN).

There are a lot of data that demonstrate Tat and Nef effects on cellular metabolism. It has been shown that these regulatory proteins can be influenced by the process off proliferation (13, 14, 15, 16, 17) apoptosis (18, 19) cancerogenesis (17, 20, 21) sensitivity cells to ionizing radiation (22) neurotoxicity (23) and some others.

The study of HIV regulatory genes attaching the cells which are nonpermissive for the virus is a way to estimate the universality of these genes action on metabolic processes in animal cells. The use of pluripotent embryonic stem cells for this purpose also allows to investigate the influence of the viral genes not only on the cell proliferation but on cellular differentiation as well.

The goal of this work is to study the effect of these viral regulatory genes on ESC proliferation, EBs formation and their differentiation in cardiomyocytes, neuronal and glial cells *in vitro*.

## MATERIALS AND METHODS

### Growth of ESC and embryonic fibroblasts

Mouse R1 line ESC (kindly presented by Dr. A. Nagy, Mount Sinai Hospital, Canada) was isolated from agouti-color (129/Sv × 129/SvJ) F1 line mouse blastocytes (24). R1 line of ESC was routinely maintained at 37°C humidified air with 5% CO_2_ in alfa-MEM medium containing 15% fetal bovine serum (FBS) (“Gibco”, USA), 0.1 mM 2-mercaptoethanol, 2 mM L-glutamine, nonessential amino acids (“Gibco”, USA), nucleosides (“Sigma”, USA), vitamins (“ICN”, USA) and gentamycin (20 μg/ml). As a nutrient (feeder) layer for ESC, primary fibroblasts from 11-12 day-old embryo mice whose proliferation had been blocked by mitomycin C (5 μg/ml) were used. DMEM (“Sigma”, USA), containing 10% of FBS and all above mentioned additions except 2-mercaptoethanol, was used as the growth medium for the primary culture of fibroblasts. When ESC were cultivated without feeder, LIF (leukemia-inhibiting factor) (“Sigma”, USA) at a concentration of 10 ng/ml was added to the medium to block spontaneous differentiation of these cells (25). Cell replating accompanied by medium changing was repeated every three day.

### Recombinant plasmids

Plasmid *pNEF* containing the *nef* gene of HIV-1 HXB3 isolate (the *Hind*III/*Taq*I-fragment, nucleotides from 8306 to 8998) under the control of cytomegalovirus (CMV) promoter was generously supplied by Dr. Hammes (26). To construct a HIV gene *tat*-carrying plasmid, the *SalI/PstI* fragment (from nucleotide 5786 through to the 3’ end) of HXB2 isolate with the deleted gene *nef*-carrying region (the *Bam*HI/*BgI*II fragment, nucleotides 8475 to 9558) was used. The *Sal1/Pst1* fragment containing 2^nd^ and 3^rd^ exons (the 1^st^ and the 2^nd^ translated exons, respectively) of the gene *tat* and the polyadenylation signal on its own was cloned after blunting the ends in the expression vector *pcDNA3* (“Invitrogen”, USA) at the *EcoR*V site. The obtained plasmid, *pTat-neo,* carried the gene of resistance to neomycin and contained gene *tat* under CMV promoter. Recombinant plasmids were purified by standard method (27).

To produce ESC transfected by the *eGFP* gene, we used the plasmid *pEGFP-C1* (“Clontech”, USA, No 6084-1) carrying this gene under the control of CMV promoter. *eGFP*-transfected cells were observed under a fluorescence microscope Axioskop 2 plus (“Carl Zeiss”, Germany).

### Cell transfection and selection

ESC transfection by the vectors containing the *tat* or *nef* genes was performed by electroporation on “SUM-4” electroporator (Institute of Molecular Biology RAS, Moscow, Russia) under the following specially selected conditions: impulse duration, 1.5 ms; power, 400 W. 10 μg of plasmid DNA was used for the transfection of 1,000,000 cells. To reveal transfectants with the *nef* gene, co-transfection of *pNef* plasmid with plasmid *pSV2neo* (kindly presented by Dr. Southern P.J.) at a ratio of 10:1 was performed. ESC were transfected also by plasmid *pSV2neo or pcDNA3* alone and used as a control. The selection of transfected cells in both cases was performed using G418 (200 μg/ml). The frequency of transfection was near 2 × 10^-4^. Polyclonal cultures were used in all further experiments. Every three months additional selection with G418 was performed for all transfected cell cultures to prevent the appearence the cells without transgenes.

The presence of the *nef* and *tat* gene transcripts in transfected cells was analyzed by RT- PCR with “Access RT-PCR System” (“Promega”, USA). The following primers were used:

for the *nef* -

5’-GGTGGCAAGTGGTCAAAAAGTAGTG-3’,
5’-AATCAGGGAAGTAGCCTTGTGTGTG-3’

for the *tat* -

5’-GAGCCAGTAGATCCTAGACTAGAGC-3’
5’-TCTGATGAGCTCTTCGTCGCTGTC-3’

Conditions for PCR were the following: 94°C 48 s., 59°C 30 s, 72°C 1 min., 27-30 cycles, then 72°C 7-10 min. The predicted sizes of amplicons were: for the *tat* - 177 bp, for the *nef - 367* bp.

### Assay for cell proliferation

An assessment of the proliferative activity of control and transfected ESC lines was carried out by the cytochemical method using MTT (3-(4,5-dimethyl tiasole-2-il)-2,5-diphenyl tetrasolium bromide) as previously described (28). At the same time, the number of alive cells used in the experiments was evaluated by Trypan Blue exclusion test under the inverted microscope Olympus CKX41 (“Olympus”, Japan) in a hematocytometer. The results were taken on the 3^rd^ day after the cell plating.

### EBs formation and cardiomyocyte differentiation

To perform the differentiation of ESC into EB, ESC was separated from fibroblast feeder. For this purpose, the cells were treated with trypsin (0.05%, 5 min) and obtained cell suspension were preplated on Petri dish (“Nunc” Denmark, d=60 mm) during 15 min at 37°C, 5% CO_2_. This time was sufficient for the main mass of fibroblasts to get attached to the bottom of the plate, whereas ESC was stayed in the suspension. Cells from the suspension were pelleted by centrifugation, and viable cell numbers were determined. The frequency of contaminating embryonic fibroblasts in the undifferentiated (day 0) ESC samples was estimated to be less than 0.2% based on cell size during counting. To form EBs, suspension of ESC (1000 cells per well) was transferred to 96-U plates (“Nunc” Denmark) and then they were placed in CO_2_-incubator (5% CO_2_). After 3 days the formed EBs were counted in 10 wells for each cell line. The quantities of the beating clusters of cardiomyocytes in the plates were counted during 3 weeks of the incubation under the inverted microscope Olympus CKX41 (“Olympus”, Japan). Each experiment was repeated at least 4 times.

### Neuronal and glial differentiation

EBs obtained from ESC were incubated for 2 days in the culture medium containing 1 μM of retinoic acid and then seeded by 8-10 EBs per well on gelatin (0.01%) coated 24 well plates (“Nunc” Denmark). The same quantities of EBs were incubated without retinoic acid and served as a control. Plates were placed in CO_2_-incubator (5% CO_2_) for differentiation for 20 days. Half of the culture medium was changed every 3 days. To detect different types of ESC differentiation, 10 days later part of the plates were processed for immunocytochemistry with antibodies raised against nestin, an antigen for neuronal precursor cells (“Chemicon”, USA) and GFAP (glial fibrillary acidic protein) (“Sigma”, USA). The remaining plates were incubated for additional 10 days to obtain neuronal differentiation which was detected with antibodies to MAP2 (mature neuronal phenotype) (“Sigma”, USA). To obtain quantitative characterization of neuronal differentiation of transfected cells the experiments were performed as follow. 8-10 EBs obtained from each of the cell line were transferred to the well of 24-well plate and after 10 days (for nestin) and 20 days (for MAP2) of incubation the quantities of nestin- and MAP2-positive cells were determined in each well using the microscope Olympus CKX41 (“Olympus”, Japan), as described below (see “Materials and Methods”, Immunocytochemistry). Ten wells for each cell line were counted.

### Tests on pluripotency of transfected ESC

**Alkaline phosphatase detection.** ESC was plated onto cover glasses with a mitomycin-blocked fibroblast feeder. After one day of cultivation, the cells were fixed by cooled acetone (+4°C) for 1 min, air dried and then incubated in a solution of AS-B1-phosphate and BB-blue dye at 37°C for 1 h (29). The stained cell preparations were studied under the microscope, and cells producing a positive response to alkaline phosphatase activity were regarded as non-differentiated.

**SSEA-1 detection.** The colonies of ESC grown on fibroblast feeder were washed two times with PBS and fixed in 100% methanol (-20°C, 5 min). Then cells were washed two times by PBS-0.01% Tween-20 and nonspecific binding was blocked by PBS-0.01% Tween-20 solution containing 5% of dry milk (solution A) and 15% FBS at room temperature for 30 min. Cells were incubated with anti-SSEA-1 mouse monoclonal antibodies (1:10 in solution A) for 1 h at room temperature. After 3 times washing with PBS-0.01% Tween-20 the secondary Alexa Fluor® 546 goat anti-mouse IgG (H+L) (1:1000) from “Molecular Probes” (USA) in PBS-0.01% Tween-20 were added for additional 1 h. After incubation at room temperature samples were washed 3 times with PBS-0.01% Tween-20 and investigated under Axioskop 2 plus microscope (“Carl Zeiss”, Germany).

#### Immunocytochemistry

The cells generated from EBs were fixed with freshly prepared 4% formaldehyde in PBS during 30 min. After 4-time washing with PBS, cells were incubated in PBS with 0.1% Triton X-100 and 2-5% of FBS (solution B) during 15 min. Then the samples were incubated with the first specific antibodies (anti-nestin 1:500, anti-GFAP 1:100, anti-MAP2 1:500) overnight at +4°C. Next day antibodies were removed by three times washing with solution B and incubated at room temperature for additional 2 h with biotinylated secondary antibodies against mouse immunoglobulins (1:500; “IMTEK”, Russia). After three times washing with solution B streptavidin conjugated with peroxidase (1:100; “IMTEK”, Russia) was added for 1 h. After the incubation and washing (three times with solution B) colour was developed with 3-amino-9-ethylcarbazole (“Sigma”, USA). The detection of stained cells in cultures was performed using the microscope Olympus CKX41 (“Olympus”, Japan).

### Statistical analysis

All statistical analyses were performed using the “Sigma Plot” software package (“Jandel Scientific Corp.”, USA). Results are expressed as mean ± SEM. Differences between groups of data were analyzed by one-way analysis of variance (one-way ANOVA). Statistical significance was accepted if *p*<0.05.

## RESULTS

### Gene expression under CMV promoter

**Figure 1:**
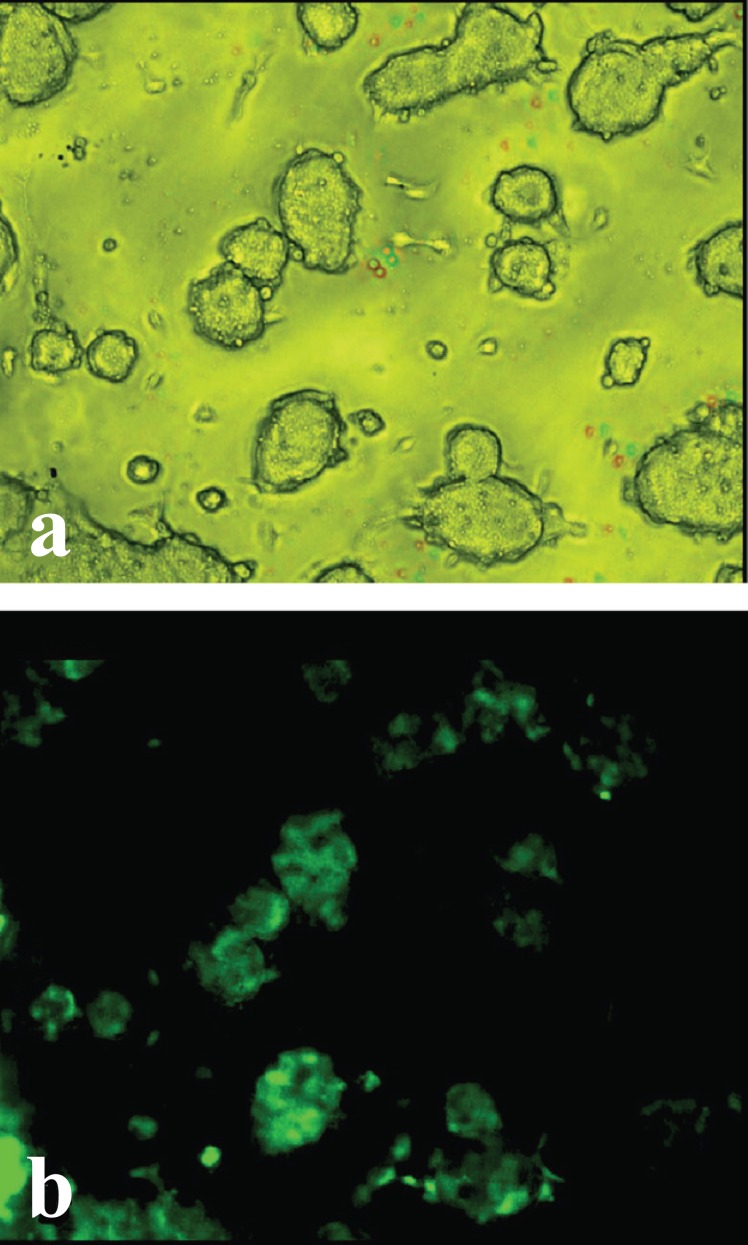
EGFP expression in ESC transfected by a plasmid carrying the *eGFP* gene under the control of CMV promoter. a, colonies of ESC in a light microscope; b, the same colonies in a fluorescent microscope; magnification ×200.

The efficiency assessment of the CMV promoter for transgene expression in ESC line R1 was conducted at the first stage of our work. The necessity of this kind of experiment was started in light of a study showing weaker activity of the CMV promoter in ESC lines D3 and J1, as compared with to CBA and EF promoters (30). The results of our experiments (Fig. 1) showed that the expression of *EGFP* gene under the control of the CMV promoter in R1 line of ESC was efficient.

The data obtained allowed us to take under the experiments on the ESC transfection by constructs with the regulatory *tat* and *nef* genes of HIV-1 under the control of the CMV promoter.

### Characterization of transfected cell lines

**Figure 2:**
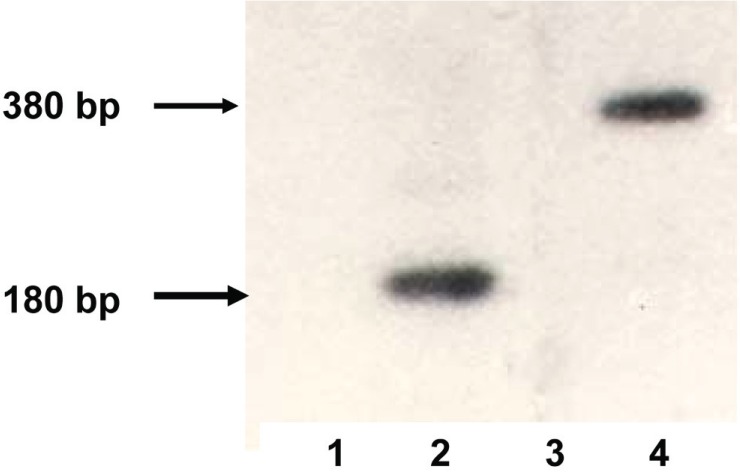
*tat* and *nef genes* expression assessed by RT-PCR. Lanes 1,3, cell line es-neok (negative control), lane 2, cell line es-tat, lane 4, cell line es-nef.

After the transfection of ESC and subsequent selection four different cell lines were obtained: es-neok (transfected by plasmid *pSV2neo*, control), es-pcDNA3 (transfected by pcDNA3, control), es-tat (transfected by plasmid *pTatneo*) and es-nef (transfected by plasmids *pNef* and *pSV2neo*). By RT-PCR expression of the *tat* gene in es-tat cells and the *nef* gene in es-nef cells was detected (Fig. 2).

**Figure 3:**
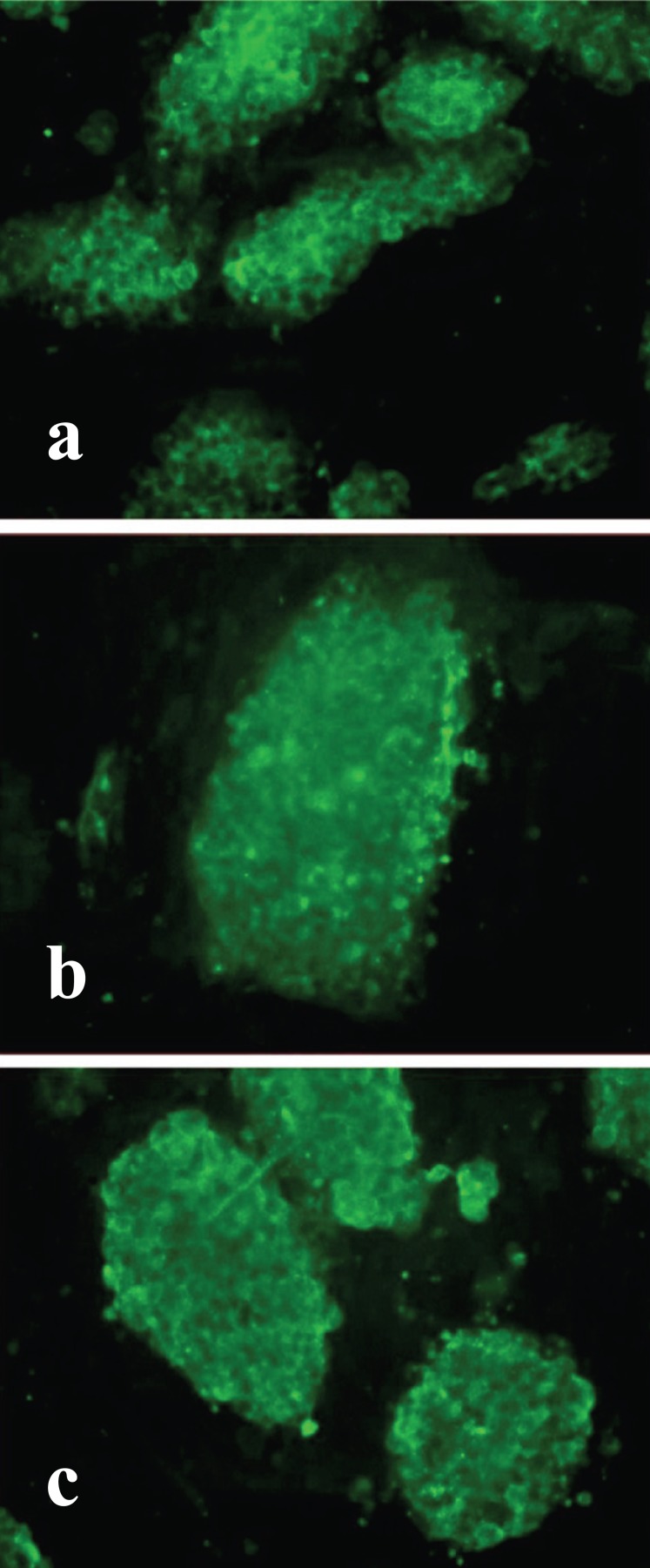
Immunofluorescent detection of SSEA -1 expression in transfected ESC lines. a, es-neok (control); b, es-tat; c, es-nef; magnification ×200.

**Figure 4:**
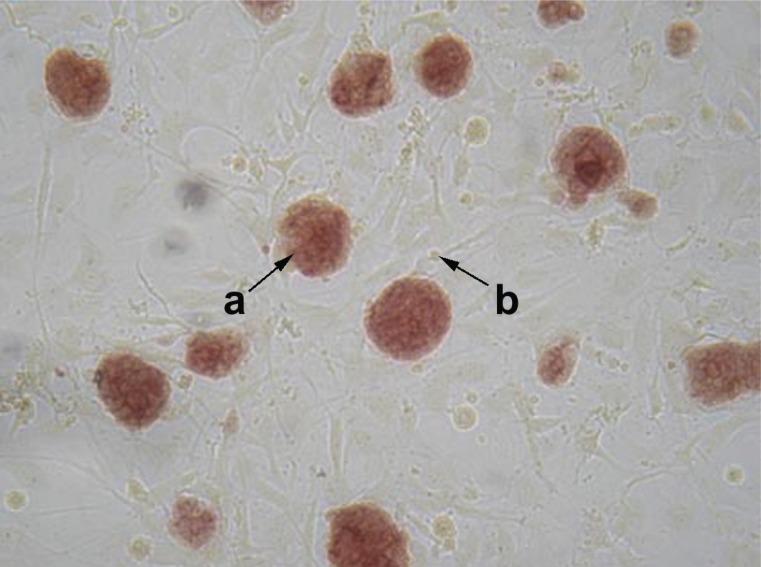
Detection of alkaline phosphatase in transfected ESC growing on fibroblast feeder cells. a, colonies of es-nef, stained; b, fibroblast feeder cells, not stained (date for es-neok and es-tat not shown); magnification ×200.

No changes in morphology were observed in each of the four ESC lines. The alkaline phosphatase and SSEA-1 staining served as markers of the mammalian ESC pluripotent status (25, 30, 31, 32). All the obtained cell lines had the same level of staining of these markers (Fig. 3 and 4).

Although the expression of the *nef* gene in the es-nef cell line was easily detected in the first passages, it was entirely lost at later passages. This result is in agreement with the data obtained earlier and the observation that it is difficult to establish stable cell lines constitutively expressing Nef due to its toxicity in transfected cells (33, 34)

### Influence of the tat and nef genes on proliferation of ESC

**Figure 5:**
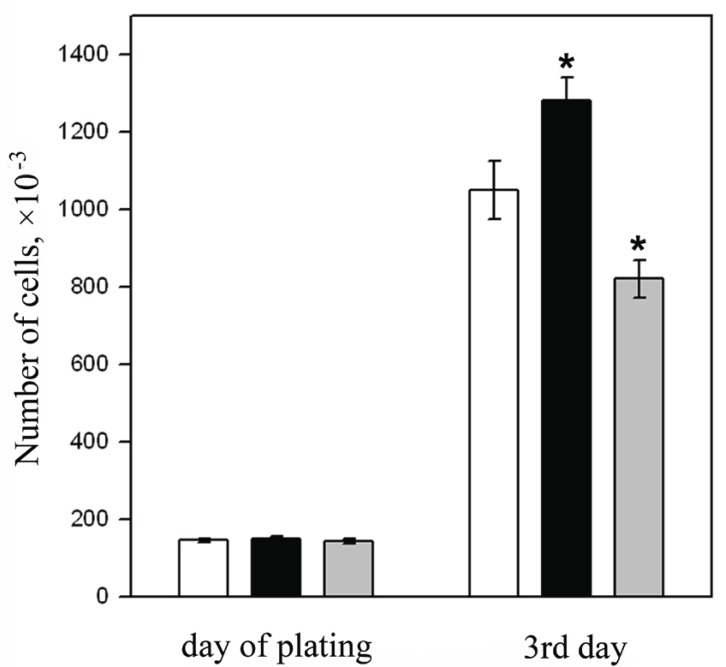
The effect of HIV-1 regulatory genes *tat* and *nef* on the proliferation of transfected ESC: white column-es-neok (control), black column-es-tat, gray column - es-nef. _*_p<0.05, n=20.

Differences between the lines of ESC transfected by the *tat* and *nef* were brought to light when studying their proliferating activity. As follows from the results presented in Fig. 5, ESC proliferation in the es-tat cell line (black column) was about 20% higher and in the es-nef cell line (gray column) was about 20% lower compared to the es-pcDNA3 and es-neok control (white column) cell lines respectively.

There were no reliable differences in proliferative activity between control cell lines es-neok and es-pcDNA3 in our experiments. The results with only es-neok line are presented in Fig. 5. Transfection of the plasmid containing the *tat gene* under the control of metallotioneine promoter (16, 17) led to the same effect (data not shown).

### Influence of tat and nef genes on EBs formation and their differentiation into cardiomyocytes

The next stage of our work was the investigation of the effect of *tat* and *nef* genes on spontaneous formation of EBs *in vitro*. EBs derived from ESC involves many events of early embryonic development. The analysis of spontaneous differentiation showed that EBs was formed after 2 or 3 days of ESC plating in the absence of the feeder cells. When EBs was transferred onto gelatin coated 4 well plates it was attached to the substrate so that the migration of the cells from the EBs and their differentiation into the cells of different types were observed. Our experiments allowed to see that the efficiency of EBs formation in R1 cell line was dependent on the number of cells, seeded on the feeder-free surface of the plate and the cultivation time of EBs before the beginning of differentiation.

After 5-6 days of EBs attachment to the surface, spontaneously contracting cardiomyocyte cells were observed. The time of cardiomyocyte development *in vitro* varied from 7 to 20 days after cell plating. The pulsation frequency of these cells was also variable and dependent on cultivation time of EBs. The frequency did not exceed 60 contractions per minute during the first day and slowly decreased eventually.

When analyzing a possible effect of *tat* and *nef* genes on ESC differentiation, experiments were conducted to estimate the following parameters for all cell lines: 1) time needed for EBs formation, 2) the number of formed EBs after 3 days of cell plating, 3) EB’s size, and 4) the occurrence of the cells with different tissue specificity (in our case we assessed ESC differentiation into cardiomyocytes). It was demonstrated that the time of EBs formation was similar for all ESC cell lines (es-neok, es-pcDNA3, es-tat, and es-nef). No significant differences in EBs sizes in these lines were revealed either. However, the number of the EBs was different: it was approximately 2 times less in the es-tat cell line than in the es-pcDNA3 line, whereas, some increase (approx. 1.4 time) in the number of EBs was observed in the es-nef line as compared to the es-neok cell line (Table 1). Again no differences in the quantity of EBs between two control cell lines were observed.

**Table 1:** Influence of regulatory genes HIV-1 *tat* and *nef* on EBs formation and differentiation in cardiomyocytes in transfected ESC

Transfected cell line	The number of EBs bodies after seeding 1000 cells per well (3 days *in vitro*)	The number of clasters of cardiomyocytes (calculated on 200 EBs)

es-neok (control)	10.00 ± 0.80	5.0
es-tat	6.27 ± 0.33^b^	3.0
es-nef	14.05 ± 1.80^a^	11.0

a *p*<0.05, n=40;

b *p*<0.01, n=40.

The areas of contracting cardiomyocytes started appearing on the 5-6^th^ day after the EBs attachment to the carrier in the es-tat and es-nef ESC lines. The amount of EBs with the clusters of contracting cardiomyocytes was higher in es-nef cell line, and it was considerably lower in the es-tat cell line against control cells (Table 1).

### The effects of the tat and nef on ESC differentiation into glial and neuronal cells

The influence of the *tat* and *nef* genes of HIV-1 on the differentiation of mouse ESC into neuronal and glial cells in the presence and absence of retinoic acid was studied. Retinoic acid (RA) is well known inducer of neuronal differentiation (35, 36). To study neuronal and glial differentiation EBs were incubated in the presence or absence of retinoic acid as described in “Material and Methods” and transferred to 4 well plates coated with 0.01% gelatin. After 10 days part of the wells were stained with antibodies to GFAP, nestin or MAP2.

No significant differences in the number of GFAP-positive cells were observed between all cell lines in the presence and absence of retinoic acid. Thus, both viral regulatory genes did not essentially affect the differentiation of ESC into glial cells.

The number of nestin-positive cells was unchanged in the *tat* gene transfected cells, but it was decreased to none in nef-transfected cells (Table 2). Therefore, the *nef* gene apparently suppressed neuronal differentiation of ESC. However, in the presence of retinoic acid the number of nestin-positive cells was greater in es-nef as compared with es-tat and control cell lines (Table 2).

**Table 2:** Influence of retinoic acid (RA) on neuronal differentiation of ESC transfected by the *nef* and *tat* regulatory genes of HIV-1

Antibodies	Transfected cell lines	The number of stained cells per well	
		-RA	+RA

**Nestin**	es-neok (control)	62±25	304±48
	es-tat	54±21	336±52
	es-nef	1-2	535±65
**MAP-2**	es-neok (control)	9±2	100±10
	es-tat	6±4	120±15
	es-nef	No	250±22

Terminally differentiated neuron cells were stained with antibodies against MAP2. In contrast to the *tat gene* transfected and control cells, the cultures of the *nef* gene transfected ESC in the absence of retinoic acid completely lacked MAP-positive cells. In the presence of retinoic acid, MAP2-positive cells could be observed in all cell lines, but the number of these cells being 2-2.5 fold higher in es-nef cell line as compared with es-tat cell line and control cells (Table 2).

## DISCUSSION

An increase in proliferating activity was observed in the mouse ESC under the influence of the *tat* gene and its suppression under the influence of the *nef* gene. The result was consistent with our previous data obtained in different rat cell lines (Rat-2 line pseudonormal cells and pheochromocytome cells line PC-12) (17).

Data obtained in this work is shown for the first time that both viral regulatory genes affect not only ESC proliferation, but have the profound effects both on early stages of ESC differentiation (EBs formation) and on the processes of their differentiation into cardiomyocytes and neuronal cell types.

The effects of the *tat* and *nef* genes on ESC were apparent as early as at the stage of EB formation, and they were strictly opposite. Whereas *tat* hampered the EB formation, the *nef* gene significantly stimulated this process. The impact of these genes was detected also at later stages of ESC differentiation.

HIV-1 cardiomyopathy has become one of the major causes of death in AIDS patients, but its pathogenesis is unclear. Here, we have for the first time shown that the expression of the *tat* gene reduced spontaneous differentiation of ESC into cardiomyocytes. The Tat protein of HIV-1 is a powerful activator of viral genes expression. Besides this essential function of the HIV-1 promoter, the Tat also exerts a remarkable number of other biological activities including the effects on cell proliferation, differentiation and cell death (37). Moreover it has been shown that Tat can induce apoptosis in different cells, including cardiomyocytes (38). Our results showing the suppression of cardiomyocytes formation in *tat*-transfected cultures are in agreement with these data.

From the other hand, it is well known that Tat protein has been suspected of causing neuronal disfunction that often leads to the development of HIV-associated dementia in AIDS patients. The treatment of neuronal cells with Tat affects the nerve growth factor (NGF) signaling pathway (39). However, we did not find any essential influence of *tat* on the processes of neuronal or glial differentiation *in vitro*, using the mouse ESC model.

The effects of the *nef* gene on ESC are generally opposite to those of the *tat* gene. We have shown earlier that the *nef* gene decreased cell proliferation in different rat cell lines *in vitro* (16, 17). In the present work we have shown that proliferation of ESC was also decreased in the *nef-*transfected cell line. Moreover, our results allowed us to reveal a new potential of the *nef* gene. It was found to induce spontaneous differentiation of ESC cells into EB and cardiomyocytes, but at the same time to completely inhibit spontaneous neuronal differentiation. The latter is in agreement with earlier data that Nef can mediate neural cell death (12, 23, 40, 41). The *nef* gene does not express in neurons of HIV-infected patients, therefore, some observed pathological effects in nervous system may be due to its expression in glial cells.

Unexpectedly, the *nef* gene does not suppress the retinoic acid induction of ESC differentiation into neurons, it can even slightly stimulate this process. It might be due to a possible anti-apoptotic effect of Nef (42) on neuronal cells partially neutralizing the pro-apoptotic effect of retinoic acid (43) when Nef cytotoxicity is attenuated by this acid. Such a ‘neutralization’ might occur e.g. due to retinoic acid suppression of synthesis of interferon-inducible protein 10 (IP-10) (44) also involved in the Nef protein mediated neuronal cell death (12).

In common, the reverse correlation between the effects of the *tat* and *nef* viral genes on ESC proliferation and differentiation were observed. Genetic modifications of ESC is a perspective approach not only for the investigations of the role of different genes in the processes of cellular and tissue differentiation but for the creation of the models for drug testing and for development of the new approaches to human cell therapy in the future.
